# Induced Pluripotent Stem Cell-Derived Dopaminergic Neurons from Familial Parkinson’s Disease Patients Display α-Synuclein Pathology and Abnormal Mitochondrial Morphology

**DOI:** 10.3390/cells10092402

**Published:** 2021-09-13

**Authors:** Xiaojun Diao, Fei Wang, Andrea Becerra-Calixto, Claudio Soto, Abhisek Mukherjee

**Affiliations:** 1 Mitchell Center for Alzheimer’s Disease and Related Brain Disorders, Department of Neurology, McGovern Medical School at the University of Texas Health Science Center at Houston, Houston, TX 77030, USA; 15969683747@163.com (X.D.); Fei.Wang.1@uth.tmc.edu (F.W.); Andrea.d.BecerraCalixto@uth.tmc.edu (A.B.-C.); Claudio.Soto@uth.tmc.edu (C.S.); 2 Department of Neurology, Xiangya Hospital, Central South University, Changsha 410078, China

**Keywords:** Parkinson’s disease, iPSC, dopaminergic neurons, α-synuclein aggregates, Lewy bodies, mitochondria

## Abstract

Accumulation of α-synuclein (α-syn) into Lewy bodies (LBs) and mitochondrial abnormalities are the two cardinal pathobiological features of Parkinson’s disease (PD), which are associated with the loss of dopaminergic neurons. Although α-syn accumulates in many different cellular and mouse models, these models generally lack LB features. Here, we generated midbrain dopaminergic (mDA) neuronal cultures from induced pluripotent stem cells (iPSCs) derived from familial PD (fPD) patients and healthy controls. We show that mDA neuronal cultures from fPD patients with A53T mutation and α-syn gene (*SNCA*) triplication display pathological α-syn deposits, which spatially and morphologically resemble LBs. Importantly, we did not find any apparent accumulation of pathological α-syn in mDA neuronal culture derived from a healthy donor. Furthermore, we show that there are morphological abnormalities in the mitochondrial network in mDA neuronal cultures from fPD patients. Consequently, these cells were more susceptible to mitochondrial damage compared with healthy donor-derived mDA neuronal cultures. Our results indicate that the iPSC-derived mDA neuronal culture platform can be used to investigate the spatiotemporal appearance of LBs, as well as their composition, architecture, and relationship with mitochondrial abnormalities.

## 1. Introduction

Parkinson’s disease (PD) is pathologically characterized by the presence of α-synuclein (α-syn)-containing deposits in neuronal perikarya (Lewy bodies, LBs) and neuronal processes (Lewy neurites, LNs) and the loss of dopaminergic (DA) neurons in the substantia nigra pars compacta (SNpc) [1]. Several different mutations in the α-syn gene, *SNCA*, lead to autosomal dominant forms of PD [2,3,4]. Importantly, duplication and triplication of the *SNCA* gene were causally associated with severe forms of PD [5,6]. The predominant presence of α-syn aggregates in LBs and LNs along with disease-causing mutations in the *SNCA* gene substantiated the crucial role of α-syn aggregation in PD pathology. However, the molecular mechanisms of α-syn aggregate-mediated DA neuron cell loss remain unclear. Mitochondrial dysfunction has been associated with the selective vulnerability of DA neurons in parkinsonism [7]. Apart from *SNCA*, other genes, including *PINK1*, *PARKIN*, and *DJ1*, that carry PD-associated mutations, are also implicated in mitochondrial health and functioning [7,8]. However, the relationship between α-syn accumulation and abnormal mitochondrial dynamics in PD is not currently clear. In order to understand the PD pathogenesis, it is crucial to develop an in vitro PD model that simultaneously manifests these two pathophysiological events.

Comprehensive transcriptomic profiling of human and mouse DA neurons from SNpc reveals the need for human-based models to understand DA neuronal pathology [9,10]. Induced pluripotent stem cell (iPSC) technology provides a unique opportunity to model disease biology in the background of patients’ genetic makeup. DA neurons can be efficiently generated from PD patient-derived iPSCs [11]. DA neuronal cultures derived from fPD patients manifested different aspects of PD pathology (reviewed by Sahar Avazzadeh et al., 2021 (ref. [12])). Although α-syn accumulation in fPD-iPSC-derived DA neurons has been reported in multiple studies, the formation of LB-like structures in these cells has not been shown. The characteristic localization and architecture of LBs suggest a specific mechanism of LB formation. 

In the present study, we first investigated whether fPD-iPSC-derived DA neuronal cultures exhibit α-syn accumulations, with spatial and morphological resemblance to LBs. Next, we tested whether fPD-iPSC-DA neuronal cultures manifest mitochondrial vulnerability. Using a floor-plate-based differentiation protocol [11], we generated DA neurons from a healthy individual and from fPD patient-derived iPSCs, carrying *SNCA* gene triplication and A53T mutation. We carried out a thorough phenotypic analysis of these iPSC-derived DA neurons. *SNCA* triplication-derived DA neurons showed increased levels of α-syn accumulation. Importantly, α-syn accumulateswere positive for phosphorylated α-syn at residue serine 129 (pS129), a typical marker for LBs, and displayed spatial and morphological resemblance with LBs. Furthermore, fPD-iPSC-mDA neurons were also associated with abnormal mitochondrial morphology and significant vulnerability to mitochondrial damage. 

## 2. Results

Characterization of iPSC-derived midbrain dopaminergic (mDA) neurons from familial PD patients (A53T mutation and *SNCA* triplication) 

**Figure 1:**
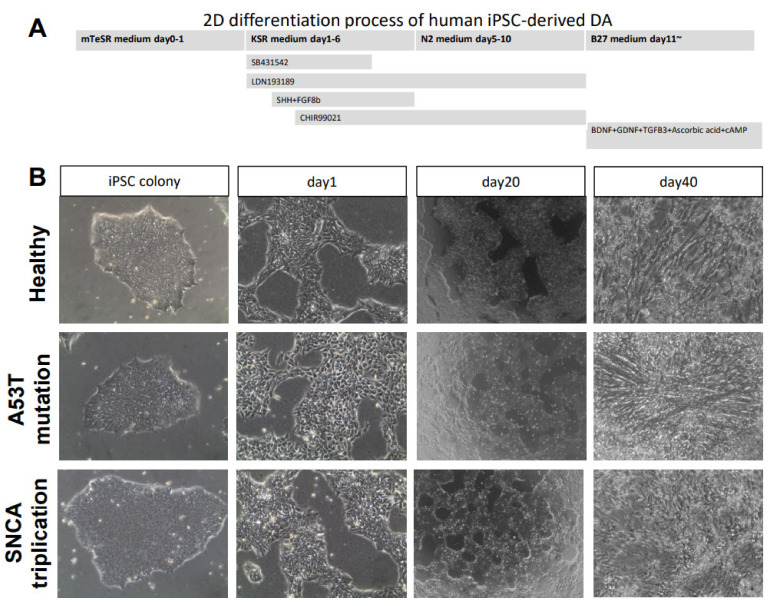
Differentiation of iPSCs to mDA neurons. (**A**) Experimental paradigm for human iPSC differentiation toward mDA neurons. (**B**) Morphology of healthy and PD patients’ cell lines at different stages during differentiation from iPSC into mature mDA neurons.

**Figure 2:**
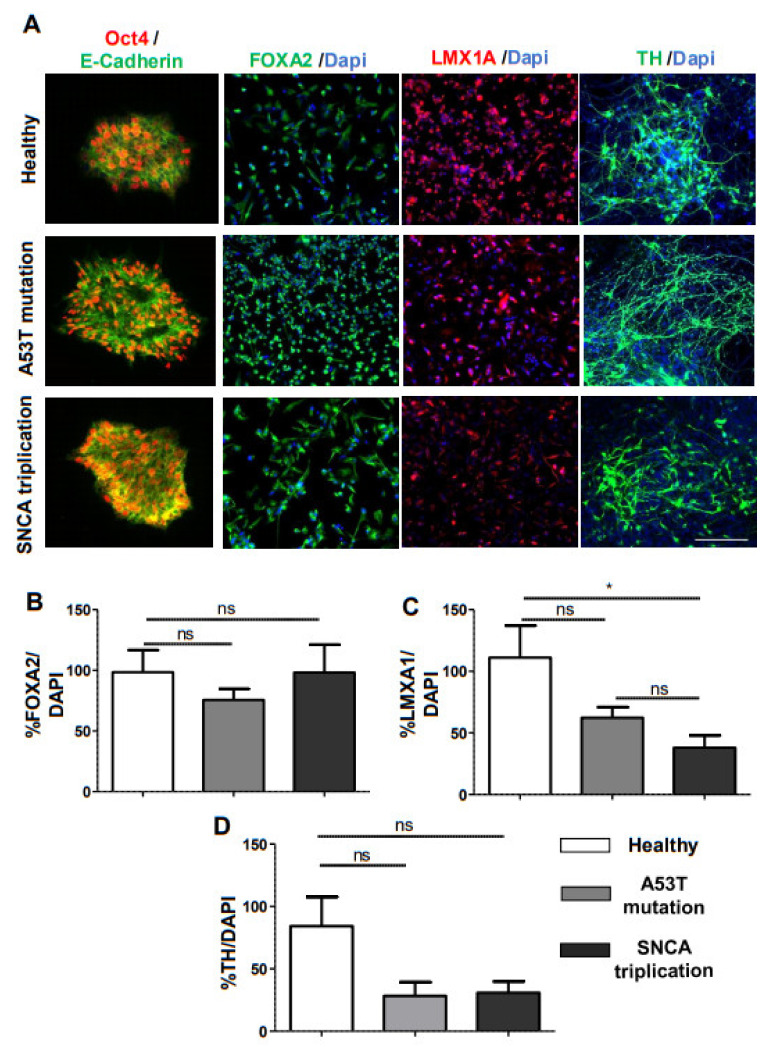
Characterization of iPSC-derived mDA neurons from a healthy subject and fPD patients. (**A**) We performed immunostaining with stem cell markers Oct4 and E-cadherin at day 0, midbrain neural precursor marker FOXA2 and LMX1A at day 11, and dopaminergic neuronal marker TH for healthy human and fPD patients (A53T mutation and *SNCA* triplication) at day 30. Next, we quantified the levels of immunostaining for (**B**) FOXA2, (**C**) LMXA1A, and (**D**) TH in healthy and fPD lines using image analysis. Images are representative of at least three independent differentiations. All data in the graphs are presented as mean ± standard error of the mean (SEM) of three images acquired from three different coverslips. Data were analyzed by one-way analysis of variance (ANOVA) followed by Tukey’s multiple comparison post hoc test. * *p* < 0.05; Scale bar is 100 µm.

In this study, we used iPSC lines from a healthy individual and two fPD patients with A53T mutation in the *SNCA* gene and *SNCA* gene triplication. To generate mDA neurons from iPSCs, we used a modified midbrain floor-plate-based mDA neuron differentiation protocol (Figure 1A) [11]. Feeder-free iPSC cultures from three different lines presented compact and flat colonies with distinct borders (Figure 1B). No obvious morphological changes among three lines of iPSC-derived mDA neurons were observed during differentiation (Figure 1B). Pluripotency was confirmed by immunostaining with Oct4 and E-cadherin (Figure 2A). At day 11 of differentiation, all the lines expressed similar levels of the Forkhead family of winged-helix transcription factor 2 (FOXA2), confirming the midbrain patterning (Figure 2A). Image quantification indicated that FOXA2 expression was present in 70–90% of the cells (Figure 2B). We noted reduced immunostaining for the LIM homeobox transcription factor 1 alpha (LMX1A) in the midbrain progenitors derived from the A53T mutant line compared to that of the healthy line (Figure 2A). However, the difference did not reach statistical significance (Figure 2C). Interestingly, there was a significant reduction in LMX1A immunostaining in the midbrain progenitors derived from the *SNCA* triplication line compared with the healthy line (Figure 2A, C). By 30 days in vitro (DIV), cells began to express the mDA neuronal marker tyrosine hydroxylase (TH) (Figure 2A). The fPD lines with A53T mutation and the *SNCA* triplication tended to have a lower percentage of TH immunostaining compared to the healthy line (Figure 2D). However, these differences did not reach statistical significance.

### 2.1. α-Syn Pathology in PD iPSC-Derived DA Neurons

**Figure 3:**
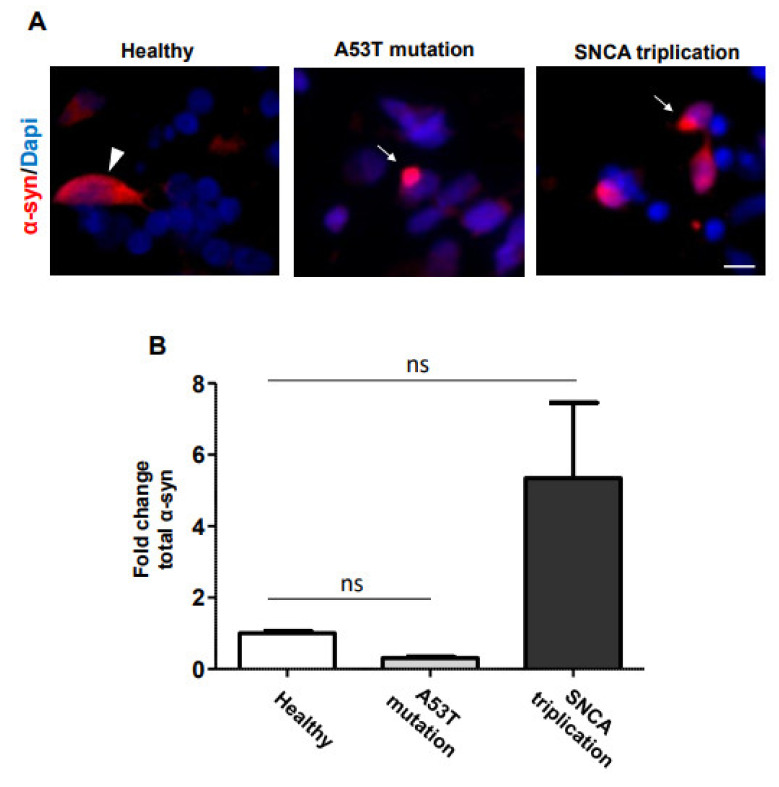
α-syn accumulation in mDA neuronal cultures from fPD patients. (**A**) Seventy-day-old mDA neuronal cultures, from healthy and familial PD lines, were stained with an anti-α-syn antibody. α-syn accumulation around the nucleus in the A53T mutation and *SNCA* triplication line is indicated by arrows, whereas the homogenous distribution of native α-syn immunostaining in the healthy line is indicated by the arrowhead. (**B**) The level of α-syn immunostaining was quantified using image analysis. Images are representative of at least three independent differentiations. All data in (**B**) are presented as mean ± SEM of three images acquired from three different coverslips. Data were analyzed by one-way ANOVA followed by Tukey’s multiple comparison post hoc test. * *p* < 0.05. The scale bar is 10 µm.

**Figure 4:**
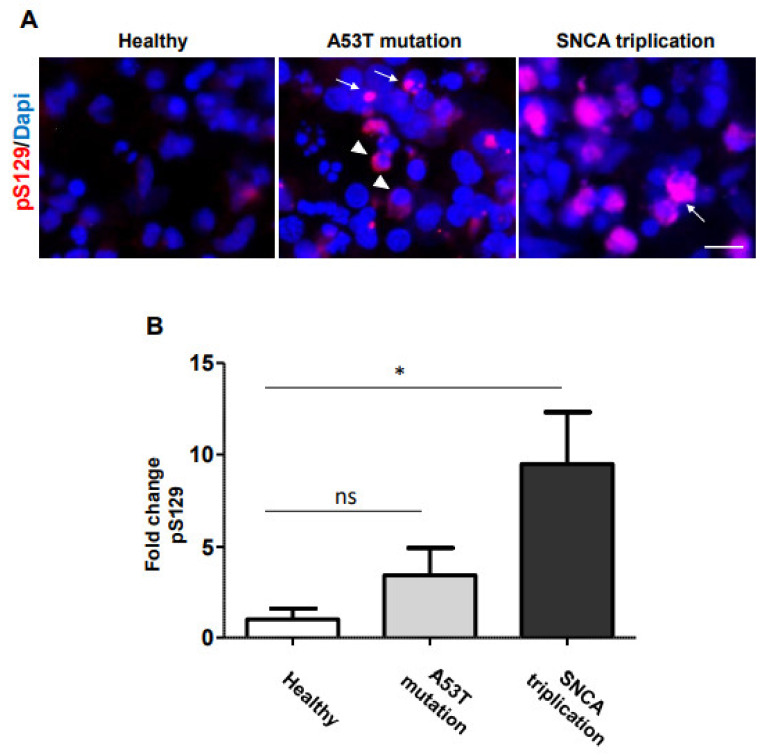
Accumulation of phosphorylated α-syn in mDA neuronal culture from fPD patient. (**A**) Seventy-day-old DA neuronal cultures from healthy and fPD lines were immunostained with an antibody against phosphorylated α-syn at serine 129 residue, a marker of LBs. The punctated accumulation of pathological α-syn next to the nucleus, resembling LBs, is indicated by an arrow, and diffuse pS129 immunostaining, resembling the early stage of LBs, is indicated by an arrowhead. (**B**) Levels of pS129 immunostaining were quantified using image analysis. Images are representative of at least three independent differentiations. All data in (**B**) are presented as mean ± SEM of three images acquired from three different coverslips. Data were analyzed by one-way ANOVA followed by Tukey’s multiple comparison post hoc test. * *p* < 0.05. The scale bar is 10 µm.

α-syn is the major component of LBs and LNs in PD^1^. In order to analyze α-syn accumulation, we immunostained the iPSC-derived DA neuronal cultures with an antibody against total α-syn at 70 DIV (Figure 3A). Image analysis indicated that mDA neuronal cultures, derived from both healthy and A53T mutant lines, have similar levels of α-syn immunostaining (Figure 3B). However, the mDA neuronal culture derived from the *SNCA* triplication line tended to show higher levels of α-syn immunostaining (Figure 3B). Importantly, in the mDA neuronal culture from A53T mutation and *SNCA* triplication line, albeit with low frequency, cells accumulated α-syn around the nucleus (Figure 3A, arrow), resembling LB formation. In contrast, healthy cells expressed a more homogeneous distribution of α-syn immunostaining in the cell body (Figure 3A, arrowhead). In order to confirm that fPD line-derived DA neuronal cultures indeed display pathological accumulation of α-syn, we immunostained the cells with an antibody against pS129, a marker of LBs [13] (Figure 4A). Healthy donor-derived DA neuronal culture rarely displayed pS129 immunostaining. In contrast, mDA neuronal culture from *SNCA* triplication line displayed significantly increased levels of pS129 compared to the healthy line (Figure 4A,B). mDA neuronal culture from an fPD patient with A53T mutation also displayed elevated levels of pS129 compared to the healthy line (Figure 4A). However, image analysis indicated that this increase did not reach statistical significance (Figure 4B). Importantly, the pS129 immunostaining in both familial PD line-derived mDA neuronal cultures appeared punctated next to the nucleus (Figure 4A arrow), resembling LBs to some extent. In some cases, the pS129 staining was diffused in the cell body (Figure 4A arrowhead) resembling the early stages of phosphorylated α-syn accumulation [14].

### 2.2. Vulnerability of fPD-iPSC-Derived mDA Neurons to Mitochondrial Damage

**Figure 5:**
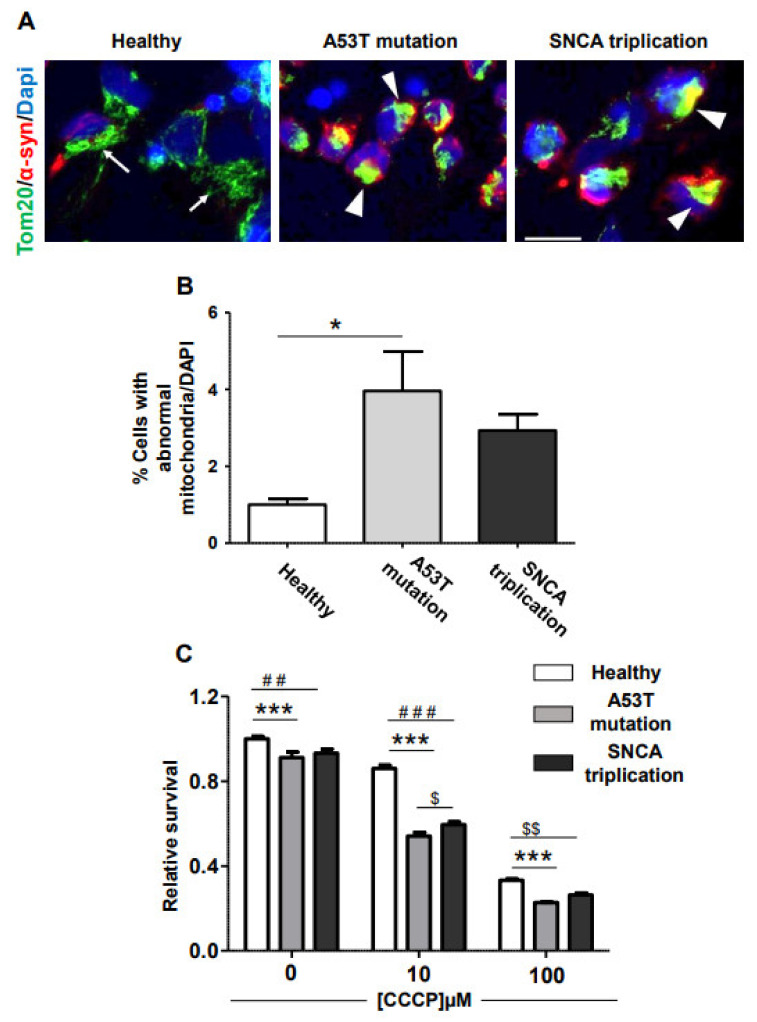
fPD-derived mDA neuronal cultures are more vulnerable to mitochondrial damage. (**A**) In order to assess abnormalities in mitochondrial morphology and its association with α-syn accumulation, we immunostained mDA neuronal cultures derived from healthy and fPD lines with an antibody against TOM20 and α-syn. mDA neuronal culture from a healthy subject presents a tubular mitochondrial network (arrow). In contrast, TOM20 staining in mDA neuronal cultures from fPD patients with A53T mutation and *SNCA* triplication appeared clumped (arrowhead), particularly in the cells with α-syn accumulations. (**B**) Abnormal mitochondrial morphology was quantified using image analysis. (**C**) mDA neuronal cultures from healthy and fPD lines were treated with different concentrations of CCCP, and cell viability was assessed using the MTT assay. All data in (**B**) are presented as mean ± SEM of three images acquired from three different coverslips. Data in figure (**B**) were analyzed by one-way ANOVA followed by Tukey’s multiple comparison post hoc test. * *p* < 0.05. All data in (**C**) are presented as mean ± SEM of four wells. Data in figure (**C**)were analyzed by two-way ANOVA followed by Bonferroni post hoc test. ## (Healthy line vs. *SNCA* triplication line at 0 µM CCCP) *p* < 0.01; ### (Healthy line vs. *SNCA* triplication line at 10 µM CCCP) *p* < 0.001; $$ (Healthy line vs. *SNCA* triplication line at 100 µM CCCP) *p* < 0.01; *** (Healthy line vs. A53T mutation line) p < 0.001; $ (A53T mutation line vs. *SNCA* triplication line at 10 µM CCCP) *p* < 0.05.The scale bar is 10 µm.

Mitochondrial damage is a common feature of PD [7]. In order to analyze mitochondrial morphology, we immunostained mDA neuronal cultures from healthy and fPD-iPSC lines with Tom20 antibody as previously described [15]. Tom20 is one of the components of the translocase of the outer mitochondrial membrane (TOM) responsible for importing mitochondrial proteins synthetized in the cytosol. mDA neuronal culture from a healthy line displayed normal mitochondrial morphology arranged in a tubular network (Figure 5A arrow). In contrast, many of the cells in fPD-derived mDA neuronal cultures manifested clump-like immunostainings (Figure 5A arrowhead), indicating abnormal mitochondrial morphology. Importantly, we also noted that clumping of mitochondrial staining tends to associate with accumulated α-syn in these cells (Figure 5A). Image analysis indicated that a significantly higher proportion of cells in the A53T line suffered from abnormal mitochondrial morphology compared to the healthy control (Figure 5B). The mDA neuron culture from the *SNCA* triplication line also displayed elevated levels of abnormal mitochondrial morphology (Figure 5B). However, this increase did not reach statistical significance. We reasoned that cells with morphologically abnormal mitochondria can be specifically vulnerable to mitochondrial damage. To test this hypothesis, we treated DA neuronal cultures from healthy and familial PD lines with carbonyl cyanide 3-chlorophenylhydrazone (CCCP) [16], a well-established uncoupler of the mitochondrial electron transport chain, and analyzed its effect on cell survival. Interestingly, even without any CCCP, the viability of mDA neuronal culture from the healthy line was higher than that of mDA neuronal cultures from familial PD lines with A53T mutation and *SNCA* triplication. The magnitude of this difference was small but significant (Figure 5C). CCCP treatment reduced cellular survival of DA neuronal cultures from all lines tested in a dose-dependent manner (Figure 5C). However, the effect of CCCP treatment was significantly higher on mDA neuronal cultures from fPD lines compared to its effect on the healthy line (Figure 5C). We also noted that the viability of the fPD line with the A53T mutation is consistently lower than that of the *SNCA* triplication line. With 10 µM of CCCP, this difference reached statistical significance. Nevertheless, in support of the mitochondrial morphological abnormalities observed in the fPD lines, cell viability data clearly indicate that mDA neuronal cultures derived from fPD lines are more vulnerable to mitochondrial damage induced by CCCP compared to those from the healthy line.

## 3. Discussion

A significant proportion of fPD-causing mutations reside in either the *SNCA* gene or genes associated with mitochondrial biology [17]. However, the mechanistic link between the *SNCA* gene product (α-syn) and mitochondrial abnormality is not completely clear. In this work, we demonstrated that iPSC-derived mDA neuronal cultures from fPD patients exhibit two crucial aspects of PD-related brain damage, the pathological accumulation of α-syn and the mitochondrial abnormalities. Importantly, we noted α-syn accumulation around the nucleus in mDA neuronal culture from the *SNCA* triplication line, spatially resembling the localization of LBs [1]. Furthermore, we observed a significant increase in the levels of pS129, one of the markers of LBs [18], in DA neuronal cultures from fPD patients with *SNCA* triplication. mDA neuronal cultures from fPD patients with A53T mutation also displayed a 2–3-fold increase in pS129 levels. However, this increase was not significant compared to DA neuronal culture from a healthy donor. The absence of LB-like α-syn accumulation is a general limitation of currently available cellular models of PD [12]. Importantly, we observed punctated accumulation of pathological α-syn (pS129 positive) next to the nucleus of cells derived from fPD lines, spatially and morphologically resembling LBs. This is a point of great interest. Recent structural analysis of α-syn aggregates revealed that aggregates spontaneously produced in a biological system can have significantly different structures compared to their in vitro-generated counterparts [19,20]. Consequently, their biology can be different. Further effort is needed to thoroughly characterize the composition and architectural features of these LB-like inclusions generated in the fPD-iPSC-derived mDA neurons using multiple independent iPSC lines from fPD patients. Evolution to fully mature LBs may require a longer time in culture. Preformed α-syn aggregates accelerate α-syn aggregation by seeding in cultured cells and animal models [21,22]. Interestingly, a recent study demonstrated that a combination of seeding with preformed α-syn aggregates and longer culture duration can lead to the formation of α-syn aggregates with biochemical, morphological, and structural features of LBs in primary mouse neuronal cultures [18]. It will be exciting to attempt a similar approach in iPSC-derived mDA neuronal cultures.

Multiple lines of evidence implicate mitochondrial abnormalities in the selective vulnerability of DA neurons from SNpc in PD. Indeed, the use of the illegal drug N-methyl-1-4-phenyl-1,2,3,6-tetrahydropyridine (MPTP), a mitochondrial toxin, led to parkinsonism [23,24]. Accumulation of somatic mutations in mitochondrial DNA during aging led to respiratory chain deficiency in the mDA neurons of SNpc [25]. Several fPD-causing mutations in genes such as *Parkin*, *PINK1*, and *DJ-1* are directly implicated in the abnormality of mitochondrial function and maintenance [7]. Our results indicate that mDA neuronal cultures from fPD patients with A53T mutation in *SNCA* gene and *SNCA* gene triplication displayed abnormally clumped mitochondrial morphology. This morphology is drastically different from tubular mitochondria arranged in a net-like structure observed in the DA neuronal culture from a healthy donor. Our observation is in line with previous reports indicating that α-syn pathology is associated with increased mitochondrial fission [15] and delayed clearance [26]. Direct interaction of α-syn with mitochondrial membranes may lead to mitochondrial fragmentation [27]. In addition to the morphological abnormalities, we also noted that clumped mitochondrial staining tends to associate with accumulated α-syn. More importantly, we showed that mDA neuronal cultures from fPD lines with A53T mutation and *SNCA* triplication were significantly more susceptible to mitochondrial damage-induced neuronal degeneration. mDA neuronal cultures from fPD patients with *Parkin* or *PINK1* mutations also display mitochondrial abnormalities associated with downstream α-syn accumulation [28]. Our results suggest that mutation and triplication of *SNCA* genes led to pathological α-syn accumulation that was associated with mitochondrial abnormalities. Taken together, these results indicate that α-syn pathology and mitochondrial abnormalities may constitute a vicious pathological cycle. Familial mutations, either in *SNCA* genes or in genes associated with mitochondrial biology, may trigger this cycle. 

PD is an age-associated neurodegenerative disease. We noted that the mDA neuron progenitors from the fPD line with *SNCA* triplication displayed reduced immunostaining for LMX1A, which is in agreement with previous findings [29]. Although the difference did not reach statistical significance, the fPD line with A53T mutation also manifested a similar tendency of reduced LMX1A immunostaining. The expression of LMX1A in fetal mDA neurons carrying fPD mutations is not known. We used two fPD lines and one healthy line in our study. It is important to validate these findings using multiple independent fPD lines and isogenic controls. LMX1A is selectively expressed in the progenitors of ventral midbrain DA neurons of mouse and plays a crucial role in their fate determination [30]. In this regard, it is important to note that neurodevelopmental defects have been recently identified in Huntington’s disease, which is normally also considered an age-associated neurodegenerative disease [31]. Likewise, Alzheimer’s disease-associated familial mutations in presenilin 1 protein caused defective neurogenesis [32,33]. Whether any neurodevelopmental defect renders the brain more susceptible to PD pathology during aging remains to be explored. 

## 4. Experimental Procedures

### 4.1. Culture of Healthy and fPD Patients’ iPSC Lines

All experimental work was approved by the Stem Cell Research Oversight Committees at the University of Texas Health Science Center at Houston, Texas, USA. iPSCs were obtained from the following donors (Table 1): (1) A 51-year-old female patient with fPD carrying A53T mutation in *SNCA* gene (NINDS Human Cell and Data Repository; cell number: ND50049) linked to early-onset PD; (2) A 55-year-old female patient with fPD carrying SNCA triplication (NINDS Human Cell and Data Repository; cell number: ND50042), and (3) A 22-year-old healthy male subject (Stem cell core at Baylor College of Medicine, Houston, Texas, USA; cell number: M22). Detailed information about the patients and cells is provided in Table 1. Feeder-free iPSC lines were routinely cultured on Matrigel (Corning) in mTeSR1 PLUS (StemCell Technologies). All iPSCs cell lines were tested for mycoplasma infection by a PCR test every month.

### 4.2. mDA Neuronal Differentiation

To generate mDA neurons from iPSCs, we used a modified midbrain floor-plate-based mDA neuron differentiation protocol (Figure 1A) [11]. Briefly, iPSCs were re-plated as single cells at density 3.6 × 10^4^ cells/cm^2^. These cells reached about 90% confluence in the plate within 24 h. For midbrain induction, we changed the medium and added DMEM with knock-out serum, complemented with small molecules SB (10 µM, TOCRIS, 1614-10) and LDN (100 nM, TOCRIS 60-531-0). This was considered day 1. SHH (100 ng/mL, R&D, 464-SH-200) and FGF8b (100 ng/mL, R&D 423-F8-025) were added from day 3 for floor plate patterning. For neuronal differentiation, N2 supplement (Gibco 17502001) was added from day 5 to day 10. A B27 supplement (Gibco 12587010) was added for the final differentiation stage starting day 11, along with BDNF (10 ng/mL, R&D 248-BDB-010), GDNF (10 ng/mL, R&D 212-GD-010), TGFb3 (1 ng/mL, R&D, 243-B3-002), ascorbic acid (0.2 mM, Sigma, 1043003), and cAMP (0.1 mM, Sigma, A6885). The cells were re-plated at day 20 for the final differentiation. 

### 4.3. Immunocytochemistry

Cells were grown on coverslips and washed 3 times with PBS before fixation. The cells were fixed in 4% paraformaldehyde for 10 minutes at 37 °C. Before immunostaining, cells were blocked with 3% BSA and permeabilized with 0.3% Triton X-100 for 1 h at room temperature. Primary antibodies were diluted in 3% BSA and incubated according to the manufacturer’s recommendations. A list of primary antibodies and sources is provided in Table 2. Appropriate Alexa Fluor 488- or 594-conjugated secondary antibodies (Thermo Fisher) were used. Finally, cells were mounted in FluorSave (EMD Millipore, 345789) with 4′,6-diamidino-2-phenylindole (DAPI, Sigma D9542) staining. Images were acquired with a Leica DMI 6000B microscope.

### 4.4. Image Analysis

Microphotographs were analyzed using Fiji-ImageJ (version 1.0 US National Institutes of Health, Bethesda, MD, USA). The images were first converted into 8-bit. The background was automatically subtracted from each image (each channel), and then we used a binary mask to obtain the relative intensity (integrated density). The relative intensity of DAPI from each image was obtained in the same way. The percentage of immunostaining was calculated by dividing the integrated density data from individual immunostaining by the integrated density of DAPI from the same image. For each line, three images at 200× magnification were quantified. To estimate the fold changes, first, the average of immunostaining from healthy cells was calculated. Subsequently, the percentage of immunostaining from individual images was divided by the average. For the mitochondrial morphology analysis, images were converted to 8-bit before thresholding (no adjustment). Then, the measurement of the abnormal mitochondrial morphology was obtained by using the “Analyze Particles” tool. The setup for size (pixel2) was 500-Infinity and for circularity was 0.30–1.00, with showing “Outlines”. The number of “Count” in the Summary was taken as the number of mitochondria with abnormal morphology. The DAPI area of the same images was measured by FIJI and used to normalize the mitochondrial abnormality analyses. The average normalized data for the healthy line was set as 1, to which the average normalized data for both A53T mutation and *SNCA* triplication lines was compared and the fold changes were plotted to generate a figure in GraphPad. For each line, three 400× images were used for the quantification.

### 4.5. Cell Viability Assay

At day 20 of mDA neuron differentiation, 5000 cells/well were plated into the pre-treated flat-bottom 96-well plate for the cell viability assay. Final differentiation was continued for 10 days. At day 30, various amounts of carbonyl cyanide 3-chlorophenylhydrazone (CCCP, Sigma C2759) were added to the differentiated mDA cultures to reach the final concentrations of 0, 10, and 100 μM. After 24 h of treatment, cell viability was measured by MTT assay (Abcam, ab211091), following the manufacturer’s instruction. Data from four replicates for each condition were used.

## Figures and Tables

**Table 1:** Information about patients and healthy control iPSCs lines.

Source	iPSC No.	Gender	Clinical Status	Age of Onset (Year)	Age at Biopsy Acquisition (Year)	Mutation
Stem cell core, Baylor College of Medicine	M22c5 (BCMi001-A)	M	Unaffected	-	22	None reported
NINDS Human Cell and Data Repository	ND50049	F	PD	39	51	*SNCA:* A53T
NINDS Human Cell and Data Repository	ND50042	F	PD	50	55	*SNCA*: Triplication

**Table 2:** List of antibodies used in this study.

Antibody	Dilution	Host	Catalog Number	Supplier
Oct4	1:1000	Rabbit	09-0023	REPROCELL
E-cadherin	1:200	Mouse	610181	BD Biosciences
FOXA2	1:200	Mouse	561580	BD Biosciences
LMX1A	1:200	Rabbit	ab139726	Abcam
Tyrosine Hydroxylase	1:1000	Rabbit	AB152	Sigma-Aldrich
Tyrosine Hydroxylase	1:1000	Mouse	22941	ImmunoStar
Tuj1	1:1000	Mouse	801201	BioLegend
α Synuclein	1:200 IF	Mouse	610787	BD Biosciences
Phospho-α-syn (PS129)	1:200 IF	Rabbit	ab51253	Abcam
TOM20	1:200 IF	Rabbit	Ab186735	Abcam

## Data Availability

The data that support the findings of this study are available from the corresponding author upon request.
